# Weighted Vests Preserve Resting Energy Expenditure During Intentional Weight Loss in Older Adults

**DOI:** 10.1093/geroni/igaf122.3868

**Published:** 2025-12-31

**Authors:** Cassidy Guida, Lauren Chandler-Holtz, Daniel Beavers, Philip Kramer, Sarah Wherry, Barbara Nicklas, Kristen Beavers

**Affiliations:** Wake Forest University School of Medicine, Winston-Salem, North Carolina, United States; Wake Forest University, Winston Salem, North Carolina, United States; Wake Forest University, Winston Salem, North Carolina, United States; Wake Forest University School of Medicine, Winston-Salem, North Carolina, United States; University of Colorado Anschutz Medical Campus, Aurora, Colorado, United States; Wake Forest University School of Medicine, Winston Salem, North Carolina, United States; Wake Forest University School of Medicine, Winston-Salem, North Carolina, United States

## Abstract

Weight regain following intentional weight loss in older adults is a near expected occurrence. The gravitostat theory suggests that gravitational loading – in the form of a weighted vest, for instance – might dampen the body’s perception of weight loss, thus mitigating signals driving weight regain, such as reduced resting energy expenditure (REE). The 12-month INVEST in Bone Health Trial (NCT04076618), compared WL alone (targeting 10% WL), WL plus weighted vest use (WL+VEST; 8 hours/day, weight replacement up to 10% total WL), or WL plus resistance training (WL+RT; 3 supervised sessions/week) on bone health in 150 older adults, is an ideal platform to examine this concept. A subset of participants (n = 21) with baseline characteristics similar to the original cohort (66.9±5.4 years; 71% women; 67% white; body mass index [BMI] 33.3±3.5 kg/m²) returned for a 24-month follow up visit (12 months post-intervention) to collect REE and body weight. At intervention end (12-months), REE was better preserved in WL+VEST [+146.9 kcal/day (95% CI: 32.4, 222.4)] compared to WL alone [-52.5 kcal/day (95% CI: -137.5, 32.5)] and WL+RT [-89.7 kcal/day (95% CI: -160.5, -18.8)]; p = 0.02. From 12 to 24 months, WL+VEST regained the least weight [+0.74 kg (95% CI:(-3.75, 5.23)], compared to WL+RT [+4.74 kg (95% CI: 0.51, 8.96)] and WL alone [+4.40 kg (95% CI (2.03, 6.77)]; p = 0.084. Findings suggest that weighted vest use may attenuate long-term weight regain in older adults by preserving REE. Future work will examine other measures of energy balance, including physical activity energy expenditure and appetite hormones.

